# A Statistical Learning‐Based Clustering Model With Features Selection to Identify Dyslexia in School‐Aged Children

**DOI:** 10.1002/dys.70013

**Published:** 2025-09-15

**Authors:** Michele Maiella, Martina Benedetti, Pierfrancesco Alaimo Di Loro, Antonello Maruotti

**Affiliations:** ^1^ Department of Behavioural and Clinical Neurology Santa Lucia Foundation IRCCS Rome Italy; ^2^ Department of Neuroscience and Rehabilitation University of Ferrara and Center for Translational Neurophysiology of Speech and Communication (CTNSC), Italian Institute of Technology (IIT) Ferrara Italy; ^3^ Department GEPLI LUMSA University Rome Italy; ^4^ Departiment of Public Health and Epidemiology Khalifa University Abu Dhabi UAE

## Abstract

The multi‐deficit framework employed to identify dyslexia requires statistical learning‐based models to account for the complex interplay of cognitive skills. Traditional methods often rely on simplistic statistical techniques, which may fail to capture the heterogeneity inherent in dyslexia. This study introduces a model‐based clustering framework, employing finite mixtures of contaminated Gaussian distributions, to better understand and classify dyslexia. Using data from a cohort of 122 children in Poland, including 51 diagnosed with dyslexia, we explore the effectiveness of this method in distinguishing between dyslexic and control groups. Our approach integrates variable selection techniques to identify clinically relevant cognitive skills while addressing issues of outliers and redundant variables. Results demonstrate the superiority of multivariate finite mixture models, achieving high accuracy in clustering and revealing the importance of specific variables such as Reading, Phonology, and Rapid Automatized Naming. This study emphasises the value of the multiple‐deficit model and robust statistical techniques in advancing the diagnosis and understanding of dyslexia.

## 1 Introduction

While reading ability remains fundamental to the identification of dyslexia, current approaches increasingly recognise it as a multidimensional condition. In this view, reading continues to play a central and indispensable role, but the inclusion of additional cognitive skills—such as phonological awareness, rapid automatized naming, visual and selective attention, auditory processing, and implicit learning—can enhance diagnostic accuracy within a multiple deficit framework (McGrath et al. 2019; Ligges and Lehmann 2022; Lorusso and Toraldo 2023). As such, dyslexia should be understood as a complex, multifactorial disorder, best captured through models that integrate multiple cognitive dimensions to improve explanatory and diagnostic precision (Elliott and Grigorenko 2014).

Within the multiple‐deficit model framework, symptoms of complex developmental disorders are thought to result from an incremental and interconnected set of dysfunctional processes (Moll et al. 2020). These theories integrate pre‐existing single‐deficit theories into a broader perspective. This calls for novel data‐analysis methods to move beyond simplistic approaches that centre on a single diagnostic phenotype, such as reading (Vellutino et al. 2004; Ramus et al. 2018), or multi‐index approaches that use weighted combinations of scores. Such approaches often produce misleading results by failing to account for heterogeneity across individuals (Giofrè et al. 2022).

Motivated by this strand of research, we analyse data published by Dębska et al. (2022), which includes information on reading, phonology, rapid automatized naming, selective attention, rhythm perception, tone comparison, visual attention span, and implicit learning. The dataset consists of 122 school‐aged children in Poland, including 51 diagnosed with dyslexia and 71 controls, typical children. As discussed in Section 2.1, this dataset has specific features requiring sophisticated modelling to avoid misleading inference. One of the aims of the work by Dębska et al. (2022) is to distinguish between children suffering from dyslexia and typical children. This was accomplished by simple univariate logistic regressions, neglecting the correlations and interactions between cognitive skills.

Recently, statistical learning approaches (James et al. 2013) emerge as key tools for accurately identifying dyslexia, as they enable the extraction of statistically significant patterns and features that characterise dyslexia's multidimensional nature. Moreover, as noted by Giofrè et al. (2019) and Eroğlu and Arman (2022), it is crucial to acknowledge the heterogeneity of data, which may stem from different subpopulations, that is, the existence of two or more clusters. Attention has, thus, shifted toward statistical and machine learning approaches, particularly clustering methods (King et al. 2007; Tamboer et al. 2016; Benedetti et al. 2022; Sharannavar and Pk 2022; Velmurugan 2023; Zingoni et al. 2024), as they offer a suitable framework to identify children with dyslexia in a multivariate, often high‐dimensional, setting. Mainly, basic clustering approaches are applied without a full investigation of the data features, ignoring the potential impact of noise, outliers, redundant and uninformative variables, which may mask the inferred clustering or bias the estimated parameters.

In this paper, we contribute to this growing body of literature. We aim to highlight the drawbacks of existing methods when outliers, redundant variables, and correlations among variables are neglected. Finite mixture models (Scrucca et al. 2023; McLachlan et al. 2019; McNicholas 2016; Peel and McLahlan 2000) represent the state of the art in model‐based clustering and the general framework we consider in this work. These models allow us to determine how various cognitive skills systematically produce signs of dyslexia in heterogeneous populations, identifying clinically interpretable variables relevant to clustering. A significant advantage of this probabilistic framework is its intuitive appeal, enabling comparisons with other methods and inference on model parameters. Furthermore, standardisation of observed variables is not required, unlike heuristic clustering methods such as k‐means, where standardisation is mandatory. Mixtures of Gaussian distributions are commonly used due to their computational and theoretical convenience, with an example of their application in dyslexia data analysis given in Giofrè et al. (2019).

In real‐world applications, data are often contaminated by outliers, noise, or spurious points. Using the terminology of Aitkin and Wilson (1980), these observations can be collectively referred to as *bad* points, while all others are *good* points. When bad points are present, they can adversely affect parameter estimation and the inferred clustering structure. To address this issue, we consider mixtures of contaminated Gaussian distributions (Punzo and McNicholas 2016; Punzo et al. 2018). A contaminated Gaussian distribution is a two‐component Gaussian mixture where one component, with a large prior probability, represents good observations and the other, with a small prior probability, represents bad observations. The latter shares the same mean but has an inflated covariance matrix, making it a simple and effective theoretical model for outliers.

Given the exploratory nature of our research question—identifying individuals with dyslexia—we adopt a flexible approach without a priori hypotheses regarding which variables to include in the clustering model. Many cognitive skill variables are highly correlated or even redundant, posing challenges for variable selection. Implicit dimension reduction techniques, such as parsimonious Gaussian mixture models based on mixtures of factor analysers (McNicholas and Murphy 2008, 2010; Maruotti et al. 2017), suggest clusters based on weighted combinations of all variables. While such techniques are valuable in many applications, they can obscure clinically meaningful subsets of variables, making results harder to interpret. Instead, we adopt a variable selection approach designed for clustering contexts, which aims to identify variables that differentiate between a priori unknown groups while eliminating those that do not. Specifically, we select variables that simultaneously minimise within‐group variance and maximise between‐group variance. This combination provides variables that best reveal group separation. Technical details of this method are provided in Andrews and McNicholas (2014).

We show how the proposed approach massively improved the clustering performance of basic approaches, widely used in the literature, further shedding light on the importance of the multi‐deficit model to correctly identify dyslexia in school‐aged children.

The rest of the paper is organised as follows. Section 2 is the core of this work. The data are introduced, with a focus on data features, in Section 2.1 and the modelling framework is briefly sketched, avoiding unnecessary technical details, in Sections 2.2 and 2.3. Comparisons among different modelling approaches, along with a discussion of the advantages of our proposal, are discussed in Section 3.

## 2 Methods

### 2.1 Data

The original dataset (Dębska et al. 2022) consists of 211 children who underwent a battery of tests assessing multiple cognitive skills. Based on their performance in reading tasks, the authors applied conservative inclusion criteria for dyslexia diagnosis. As a result, 51 children were diagnosed as having dyslexia, 71 as controls, and 81 as neither having dyslexia nor control. Eight children were considered too young to be properly diagnosed with dyslexia.

Following Dębska et al. (2022), both the ‘unclassified’ and ‘too young’ groups have been excluded from the main analysis. These groups are composed of quite heterogeneous units with little in common with one another. In particular, they might include children that do or do not have dyslexia but for which the tasks and chosen criteria were not able to provide a clear‐cut classification. Notably, it is worth exploring whether the proposed statistical method exploiting the multi‐dimensional set of cognitive skills can identify such units as an additional ‘intermediate’ group or would rather allocate each of them to either of the two original groups. This question is addressed in a secondary analysis, with the results presented at the end of Section 3.

The reading tasks employed include single‐word reading, pseudoword reading, and lexical decision tasks. Children were asked to read words of varying complexity and frequency. Then, the pseudoword test evaluated fluency by asking children to read as many pseudowords as possible from a list of 70 items within 60 s. Pseudowords were designed to be pronounceable but without close neighbours in the Polish lexicon. Children were then tasked with identifying pseudowords from a mix of 78 items (50 real words and 28 pseudowords) within 60 s, emphasising speed and accuracy. Phonological abilities were assessed using two main tests: the first one included tasks like paronym analysis, syllable analysis/synthesis, and phoneme analysis/synthesis; the second one dealt with removing specific phonemes from spoken words (e.g., saying ‘banana’ without the ‘b’). The rapid automatized naming (RAN) test assessed the speed with which children named familiar items (e.g., colours or objects) on a board containing 48 items. Visual selective attention is also measured: children identified target items (e.g., ducks with specific characteristics) from distractors within a limited time frame. In a computerised rhythm perception task, children compared pairs of short melodies to determine if they were the same or different. Melodies were designed to vary in their metrical structure and accentuation, with scores based on the percentage of correct judgments. Similarly, a computerised task evaluated pitch discrimination by asking children to identify the higher‐pitched tone between two sequentially presented tones. Visual attention span was assessed using global and partial report tasks. Children reported as many symbols as possible from a presented string. Scores were based on accurately reported symbols. Children further completed a serial reaction‐time task where they responded to visual cues on a screen by pressing corresponding keys. Implicit learning was assessed by comparing reaction times between random and structured sequences.

**FIGURE 1:**
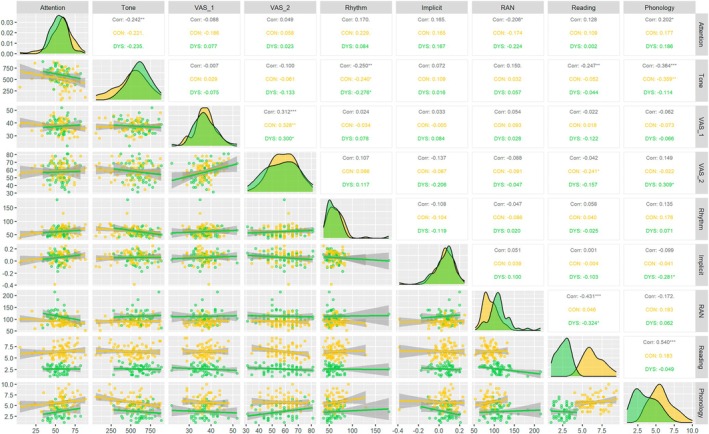
Dyslexia data features. The upper panel shows the correlation between the continuous variables, the lower panel shows the scatter plots of the continuous variables, and the diagonal shows the density plots of the continuous variables, aggregated and stratified between controls and diagnosed with dyslexia.

Figure 1 provides an overview of the key data features as multimodality, correlations among cognitive skills, and the clustering power of the variables. Differences are easily depicted between control units (in red) and units with dyslexia (in green) for a few variables (RAN, Reading and Phonology): although the conditional distributions are widely overlapping, two distinct modes can be easily depicted. For all the other variables, it is challenging to distinguish between the two clusters. This could be an indication of uninformative variables, that is, a lack of clustering power. The importance of taking into account heterogeneity is further confirmed by looking at a very simple summary measure such as the linear correlation coefficient: the marginal linear regression coefficient (in black) is sometimes statistically significant, whereas the within‐cluster correlations are not (and vice‐versa). Furthermore, the marginal correlation is often of different size than the conditional ones and could also have a different sign. Neglecting important data features, like heterogeneity, may lead to misleading statistical inference and discussions. Finally, we would like to highlight that there are a few points lying far from the bulk of the data, which may affect both the inferred clustering and the estimated parameters, potentially giving a misleading picture of the underlying data structure. Properly addressing such outliers is crucial to obtaining an accurate and robust statistical analysis.

### 2.2 Finite Mixtures of Contaminated Gaussian Distributions

The probability density function of the random vector **X** collecting the *p*‐variate cognitive skills, modelled using a finite mixture, can be expressed as:

$$p\left(x;\psi \right)=\sum_{g=1}^{G}\pi_{g}f\left(x;\vartheta_{g}\right)$$

where $\pi_{g}$ represents the mixing proportion for the g‐th cluster, satisfying $\pi_{g}>0$ and $\sum_{g=1}^{G}\pi_{g}=1$. The term $f\left(x;\vartheta_{g}\right)$ is the cluster‐specific probability density function parameterized by ϑ_g_. The parameter set ψ comprises $\pi ={\left\{\pi_{g}\right\}}_{g=1}^{G}$ and $\vartheta ={\left\{\vartheta_{g}\right\}}_{g=1}^{G}$, encapsulating all model parameters. For each cluster *g* (*g* = 1, …, *G*), this study employs a multivariate contaminated normal distribution. Its density function is defined as:

$$f\left(x;\vartheta_{g}\right)=\alpha_{g}\phi \left(x;\mu_{g},\Sigma_{g}\right)+\left(1-\alpha_{g}\right)\phi \left(x;\mu_{g},\eta_{g}\Sigma_{g}\right)$$

where *α*_*g*_ ∈ (0.5, 1) controls the proportion of data points adhering to the primary distribution, while *η*_*g*_ *>* 1 specifies the level of contamination. The parameter vector ***ϑ***_*g*_ = {*α*_*g*_, *μ*_*g*_, **Σ**_*g*_, *η*_*g*_} collects the cluster‐specific parameters. The term *ϕ*(**x**; *μ*, **Σ**) is the probability density function of a multivariate normal distribution with mean vector ***μ*** and covariance matrix **Σ**, given by:

$$\phi \left(x;\mu ,\Sigma \right)={\left(2\pi \right)}^{-p/2}{\left|\Sigma \right|}^{-1/2}\exp\left(-\frac{1}{2}\delta \left(x,\mu ;\Sigma \right)\right)$$

where *δ*(**x**, *μ*; **Σ**) = (**x** − ***μ***)^⊤^ **Σ** ^−1^(**x** − ***μ***) represents the squared Mahalanobis distance, and |**Σ**| denotes the determinant of **Σ**.

The full mixture model, integrating all clusters, can then be expressed as:

$$p\left(x;\psi \right)=\sum_{g=1}^{G}\pi_{g}\left[\alpha_{g}\phi \left(x;\mu_{g},\Sigma_{g}\right)+\left(1-\alpha_{g}\right)\phi \left(x;\mu_{g},\eta_{g}\Sigma_{g}\right)\right]$$

This approach treats the *G* clusters as distinct groups, each represented by a primary component alongside a secondary contamination component. Such a model accommodates variability within clusters while preserving robustness to outliers.

To reduce the number of parameters in the covariance matrices **Σ**_*g*_, we apply an eigen‐decomposition:

$$\Sigma_{g}=\lambda_{g}D_{g}A_{g}D_{g}^{⊤}$$

where *λ*_*g*_ = |**Σ**_*g*_|^1*/p*^ defines the overall size (volume) of the cluster, **A**_*g*_ is a diagonal matrix representing the cluster's shape via eigenvalues, and **D**_*g*_ is an orthogonal matrix specifying the cluster's orientation through its eigenvectors.

By constraining the elements of this decomposition, we have a family of simplified models with varying levels of complexity. Constraints can enforce equal or variable cluster volumes, shapes, and orientations. These simplifications allow for a trade‐off between model flexibility and computational efficiency. Table 1 outlines these 14 model configurations, along with their respective covariance structures and parameter requirements. Each model is identified by a combination of possible cluster constraints on volume, shape, and orientation, respectively. For instance, ‘EII’ specifies equal volumes, spherical shapes, and no orientation parameters, while ‘VVV’ imposes no constraints, allowing all parameters to vary freely. This approach provides a versatile framework for clustering tasks, adapting to various data structures and levels of complexity.

**TABLE 1:** Nomenclature, covariance structure, and the number of free parameters for parsimonious contaminated normal mixture models.

Model	Volume	Shape	Orientation	Covariance structure	Number of free parameters
EII	Equal	Spherical	—	*λ* **I**	*1*
VII	Variable	Spherical	—	*λ*_*g*_ ***I***	*G*
EEI	Equal	Equal	Axis‐aligned	*λ**A***	*p*
VEI	Variable	Equal	Axis‐aligned	*λ*_*g*_ ***A***	*G* + *p* − 1
EVI	Equal	Variable	Axis‐aligned	*λ**A***_*g*_	1 + *G*(*p* − 1)
VVI	Variable	Variable	Axis‐aligned	*λ*_*g*_ ***A***_*g*_	*Gp*
EEE	Equal	Equal	Equal	*λ**DAD*** ^T^	*p*(*p* + 1)/2
VEE	Variable	Equal	Equal	*λ*_*g*_ ***DAD*** ^T^	*G* + *p* − 1 + *p*(*p* − 1)/2
EVE	Equal	Variable	Equal	*λ**DA***_*g*_ ***D*** ^T^	1 + *G*(*p* − 1) + *p*(*p* − 1)/2
EEV	Equal	Equal	Variable	*λ**D***_*g*_ ***AD***_*g*_^T^	*p* + *Gp*(*p* − 1)/2
VVE	Variable	Variable	Equal	*λ*_*g*_ ***DA***_*g*_ ***D*** ^T^	*Gp* + *p*(*p* − 1)/2
VEV	Variable	Equal	Variable	*λ*_*g*_ ***D***_*g*_ ***AD***_*g*_^T^	*G* + *p* − 1 + *Gp*(*p* − 1)/2
EVV	Equal	Variable	Variable	*λ**D***_*g*_ ***A***_*g*_ ***D***_*g*_^T^	1 + *G*(*p* − 1) + *Gp*(*p* − 1)/2
VVV	Variable	Variable	Variable	*λ*_*g*_ ***D***_*g*_ ***A***_*g*_ ***D***_*g*_^T^	*Gp*(*p* + 1)/2

*Note:* Bold values indicates matrices.

### 2.3 Variable Selection

We are looking for the most relevant variables able to distinguish between controls and diagnosed children. To achieve this goal, we apply the algorithm proposed by Andrews and McNicholas (2014). The algorithm begins by computing the within‐group variance for each variable, denoted as *W*_*j*_, where *j* = 1, …, *J*. The variable that results in the smallest within‐group variance is automatically included in the initial clustering set. Subsequently, additional variables are added to the clustering set based on two criteria: their ability to separate clusters and their correlation with variables already in the set. A dynamic selection criterion is applied to facilitate this process.

This selection criterion starts with a linear relationship between the within‐group variance *W*_*j*_ and the correlation *ρ*_*jr*_ and progresses to a quintic relationship. A variable *j* is added to the clustering set *V*_*i*_ if, for all *r* = 1, … *J* in *V*_*i*_, the following condition is satisfied:

$$\left|\rho_{\mathrm{jr}}\right|<1-W_{j}^{i}$$

As the exponent *i* increases, the correlation threshold is relaxed, allowing for higher correlations between selected variables. Five exponent values are tested, *i* = 1, 2, …, 5, resulting in five potential subsets of selected variables. Model‐based clustering is then performed on each of these subsets. The final subset is chosen based on the criterion of minimising clustering uncertainty, ensuring the most robust and interpretable clustering solution.

## 3 Results

We first compare different clustering methods (hierarchical, partitioning, univariate and multivariate model‐based) and draw conclusions on the risk of obtaining misleading results when the data features are not fully investigated.

The starting point is an agglomerative hierarchical clustering algorithm, commonly used to group units in clusters based on their similarity since it is suitable for constructing and identifying typological groupings (examples on their use in analysing dyslexia data are given in Benedetti et al. (2022)). We then move to partitioning methods, focusing on k‐means and k‐medoids. All these approaches allow for spherical clusters only, and ignore potential correlations among variables; furthermore, they require a preliminary standardisation of the data.

We finally consider finite mixtures of Gaussian distributions which are the golden standard in clustering dyslexia data (see Giofrè et al. (2019) for an example in dyslexia data analysis). To investigate the multiple‐deficit model, we compare the univariate models, that is, modelling one variable at the time, with the multivariate model, where outliers are accounted for and variable selection is applied.

Comparisons among these approaches are based on the adjusted Rand index (Hubert and Arabie 1985), which has been shown to be the most desirable index for measuring cluster recovery (Milligan and Cooper 1986; Saltstone and Stange 1996; Steinley 2004). Since the true clustering is known, that is, we work under a supervised framework, we compare it with that inferred by each method. It quantifies the similarity between two partitions of a dataset by comparing the assignments of data points to clusters.

Table 2 summarises the ability of the different models to recover the true clustering structure. The closer the adjusted Rand index is to 1, the better the inferred clustering is. The results show that non‐model‐based methods perform poorly, and multivariate finite mixtures should be preferred. Focusing on finite mixture approaches, the univariate analysis allows us to draw some preliminary conclusions about the role played by the different cognitive skills in the identification of dyslexia. The negative adjusted Rand index values indicate that the agreement is less than what is expected from a random result and are a clear indication of poor classification performances. This is observed for the following variables: attention, tone identification, the two visual attention span variables, and implicit learning. Conversely, Reading is the most relevant variable in terms of clustering power, confirming its central role in the identification of dyslexia.

**TABLE 2:** Goodness of clustering.

Method	Adjusted rand index
Hierarchical clustering—Euclidean distance—Average method	0.001
Hierarchical clustering—Euclidean distance—Ward method	0.005
Hierarchical clustering—Euclidean distance—Single method	0.006
Hierarchical clustering—Euclidean distance—Complete method	0.423
Kmeans	0.696
Kmedoids	0.236
Univariate finite mixtures of Contaminated Gaussians—Attention	−0.016
Univariate finite mixtures of Contaminated Gaussians—Tone	−0.013
Univariate finite mixtures of Contaminated Gaussians—VAS 1	−0.008
Univariate finite mixtures of Contaminated Gaussians—VAS 2	−0.007
Univariate finite mixtures of Contaminated Gaussians—Rythm	0.001
Univariate finite mixtures of Contaminated Gaussians—Implicit	−0.005
Univariate finite mixtures of Contaminated Gaussians—RAN	0.013
Univariate finite mixtures of Contaminated Gaussians—Reading	0.903
Univariate finite mixtures of Contaminated Gaussians—Phonology	0.189
Multivariate finite mixtures of Contaminated Gaussians with variable selection	0.967

*Note:* The adjusted Rand index (ARI) is displayed for a range of hierarchical, partitioning, and model‐based clustering methods. A value of 1 indicates a perfect match between the true and inferred partitions; a value close to 0 indicates a random assignment of data points to clusters, and a negative value indicates a worse assignment than the random one.

The multivariate analysis performs very well, showing the highest adjusted Rand index among all the considered models, with only one misclassified observation. This result has a two‐fold implication: first, it confirms the importance of dropping uninformative variables in a multivariate setting and, second, it reveals the presence of outliers. The EVI reparameterisation is selected, according to model selection criteria, that is, correlations between variables within cluster are null. This result adds more details to the discussion between single‐deficit vs. multiple‐deficit models. Indeed, bearing in mind that reading is the most relevant cognitive skill to be investigated with no doubt, phonology, RAN and, surprisingly, tone identification further contribute to better cluster children in the groups diagnosed with dyslexia or the typical one. However, these variables appear to add incremental effects rather than interact significantly. Whilst clusters can be easily depicted for the RAN and phonology variables (as discussed in Section 2.1 and identified by the modes displayed in Figure 1), this is far from being true when looking at the tone variable (as shown the corresponding adjusted Rand index for the univariate model). However, its left‐tail behaviour differs between typical children and those having dyslexia; this is not enough to identify the two clusters if the tone variable is modelled alone but becomes meaningful when the multivariate approach is considered.

In summary, the multiple‐deficit model should be the gold standard for analyses on dyslexia detection. Cognitive skills exhibit additive effects without significant intercorrelations, as estimated within a clustering framework. Careless application of common clustering techniques without a thorough investigation of data characteristics can lead to biased results and misleading inferences.

**FIGURE 2:**
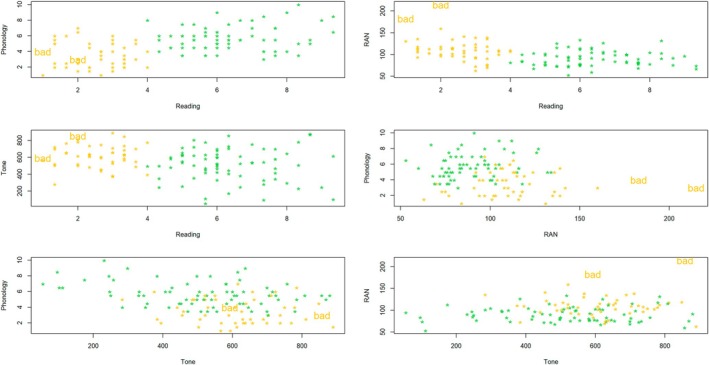
Outliers detection. For each pair of variables, we report the inferred classification. The ‘*’ denotes good observations, whilst the ‘bad’ indicates the outliers.

Finally, as a by‐product of the use of contaminated Gaussian distributions, we can identify atypical observations. Figure 2 shows the two points recognised as outliers by the model for all pairs of variables. Within the proposed framework, we do not have to discard these values in advance, but keep them in the model and handle them properly; alternative approaches exist in the literature, see, for example, Farcomeni and Punzo (2020).

It may still be of interest to analyse the dataset including the previously unclassified units for a secondary objective. Having successfully validated our model‐based procedure in distinguishing between control units and those having dyslexia, we can extend the analysis to incorporate this unclassified group and examine how their inclusion affects the clustering outcome. If additional clusters—such as a third or fourth—emerge, this would suggest that these units (or a subset of them) may form a distinct, potentially intermediate group. Conversely, if the model selection criteria continue to support a two‐cluster solution, each unclassified unit would be assigned to one of the two established groups. This outcome would imply that our model‐based approach is able to infer classifications for the unclassified units, something the original classification procedure that generated the dataset failed to achieve.

To determine the optimal number of clusters, we employed eight different model selection criteria, all of which consistently supported a two‐cluster solution. Importantly, the control units and those diagnosed with dyslexia were clearly identified, with only two cases misclassified. This finding is particularly significant, as the introduction of unclassified units could potentially introduce noise and obscure the underlying clustering structure. Instead, the robustness of the proposed approach is underscored. Among the 81 unclassified units, 43 were assigned to the group with dyslexia and 38 to the control group, with 7 observations in total identified as outliers.

## 4 Discussion

This study highlights the potential of model‐based clustering approaches, particularly finite mixtures of contaminated Gaussian distributions, in enhancing the diagnosis of dyslexia. By integrating variable selection into the clustering process, we identified cognitive skills that play a pivotal role in distinguishing between children with dyslexia and their typically developing peers. The multivariate model proved highly effective, achieving an adjusted Rand index of 0.967, significantly outperforming its competitors.

Our findings confirm the critical role of Reading as the most relevant variable in dyslexia identification, consistent with its centrality in existing literature on dyslexia diagnostics. Phonology and Rapid Automatized Naming further contributed to clustering performance, aligning with the multiple‐deficit model. Interestingly, Tone Identification also added value, although its effect was more nuanced, becoming apparent only within a multivariate framework. This result suggests that less prominent cognitive skills may still play a supportive role in identifying dyslexia when considered in conjunction with other variables.

By incorporating contaminated Gaussian distributions, the model effectively handled atypical observations without discarding them, avoiding biased results drawn if the presence of outliers is neglected.

In conclusion, this study demonstrates that robust statistical learning approaches, rooted in the multiple‐deficit model, can significantly improve our understanding and diagnosis of dyslexia. By employing advanced clustering techniques and carefully addressing data features, we provide a framework that holds promise for improving diagnostic accuracy.

## Conflicts of Interest

The authors declare no conflicts of interest.

## Data Availability

The data that support the findings of this study are openly available in OSF at https://osf.io/bp8sq.
